# Motion Smoothness Analysis of the Gait Cycle, Segmented by Stride and Associated with the Inertial Sensors’ Locations

**DOI:** 10.3390/s25020368

**Published:** 2025-01-09

**Authors:** Leonardo Eliu Anaya-Campos, Luis Pastor Sánchez-Fernández, Ivett Quiñones-Urióstegui

**Affiliations:** 1 Instituto Nacional de Rehabilitación Luis Guillermo Ibarra Ibarra, Mexico City 14389, Mexico; lanaya2021@cic.ipn.mx (L.E.A.-C.); iquinones@inr.gob.mx (I.Q.-U.); 2 Centro de Investigación en Computación, Instituto Politécnico Nacional, Mexico City 07738, Mexico

**Keywords:** smoothness analysis, gait cycle, inertial sensors’ locations, human walking, motor task, rehabilitation

## Abstract

Portable monitoring devices based on Inertial Measurement Units (IMUs) have the potential to serve as quantitative assessments of human movement. This article proposes a new method to identify the optimal placements of the IMUs and quantify the smoothness of the gait. First, it identifies gait events: foot-strike (FS) and foot-off (FO). Second, it segments the signals of linear acceleration and angular velocities obtained from the IMUs at four locations into steps and strides. Finally, it applies three smoothness metrics (SPARC, PM, and LDLJ) to determine the most reliable metric and the best location for the sensor, using data from 20 healthy subjects who walked an average of 25 steps on a flat surface for this study (117 measurements were processed). All events were identified with less than a 2% difference from those obtained with the photogrammetry system. The smoothness metric with the least variance in all measurements was SPARC. For the smoothness metrics with the least variance, we found significant differences between applying the metrics with the complete signal (C) and the signal segmented by strides (S). This method is practical, time-effective, and low-cost in terms of computation. Furthermore, it is shown that analyzing gait signals segmented by strides provides more information about gait progression.

## 1. Introduction

Human walking is an essential motor task for activities of daily living and is highly related to the quality of life [1]. The gait is an important indicator of disease progression [2,3]. Gait analysis is a valuable tool for diagnosing and monitoring the rehabilitation of various pathologies, including neurological diseases. According to González, Y et al. [4], the incidence rate of neurological diseases has increased in recent years, and the trend is expected to rise due to an aging population.

Fundamental gait analysis includes the description of its phases, the distance traveled, and speed. These parameters are easily obtained through simple timed tests. However, distance and speed are not the only variables that can describe the gait [5]. More information can be obtained through kinetic and kinematic analysis, which provides spatiotemporal parameters such as step/stride length, step width and angle, support/swing time and percentage, cadence, and speed, among others, as well as the executed joint ranges and indicators of variability, regularity, symmetry, and smoothness [1,4]. One drawback of kinetic and kinematic analysis is that it requires controlled environments and specific equipment for analysis [6], i.e., motion capture systems and force platforms [7,8]. Therefore, we must find devices and algorithms capable of continuous gait evaluation. There is a growing interest in developing methods for assessing the gait that objectively measure variables and indicators, and which can be used in daily clinical practice and scientific research [6].

The most widely used method is photogrammetric motion capture systems, considered the gold standard, tracking light-reflective markers placed on the body [6,9]. It is possible to obtain the positions of markers placed in anatomical areas within a given three-dimensional space using photogrammetric cameras and advanced digital systems [10]. However, the main drawbacks of these systems are that they must be used in controlled spaces and light conditions, and they are not very accessible due to their cost, the unavailable in many clinical facilities [10]. Another commonly used system is pressure-sensitive gait mats, which detect each step and allow quantifying spatiotemporal gait parameters [7,11]. However, analyzing the movements of the and upper extremities is impossible, and their use is mainly limited to laboratory settings [12,13].

On the other hand, force platforms are restricted to analyzing the stance phase, one foot at a time, and require complex installations. Due to their portability, miniaturization, and cost reduction, interest in using inertial measurement units (IMUs) for motion analysis has increased [14]. Since IMUs directly measure linear accelerations and angular velocities, their results are generally better than those calculated by taking the derivative of position data from photogrammetry systems [15]. Systems based on IMUs allow for sampling and studies in natural environments, measuring a comprehensive set of parameters [16], such as spatiotemporal ones and variability indicators, and even calculating a parameter called “smoothness” [17], which can reveal subtle changes even before conventional spatiotemporal parameters are calculated [18,19].

Smoothness is a metric that reflects the continuity or non-intermittency of any movement [20]. A smooth movement comprises a series of submovements closely spaced in time. In contrast, non-smooth movements result from the superposition of more submovements [17,20]; a continuous motor gesture and control are not composed of many acceleration and deceleration phases. This is a characteristic feature of healthy, well-developed, and trained human motor behavior [21]. Otherwise, for example, the movements of people with neurological diseases tend to be abrupt, with many accelerations and decelerations. As they train or rehabilitate their motor systems, they become smoother, reducing changes in acceleration [22]. There are different ways to estimate smoothness; some include Jerk calculation [23], which refers to the first derivative of acceleration and the third derivative of position, obtainable from the angular position formed by joints; the number of peaks in a normalized velocity profile; or the number of peaks in the normalized mean velocity, among others [20]. Although different metrics allow for smoothness calculation, these have mainly been used in the motor gestures of the thoracic limbs [24,25,26]. According to Balasubramanian S. et al. [26,27], most metrics, including those mentioned, do not meet the validation criteria such as consistency, sensitivity, robustness, dimensionality, monotonic response, and a low computational cost, which are necessary for the correct quantification of smoothness [27,28].

Another smoothness metric [29] is the harmonic ratio (HR). Some researchers have shown that there is a reduced value of the HR from early stages in people suffering from cognitive impairment [19] or in those with multiple sclerosis without disability [23]. The HR is a metric derived from Fourier analysis of torso accelerations. It is considered mainly an indicator of gait rhythmicity [30], with only acceleration values from an IMU placed on the torso analyzed. In 2012, Balasubramanian S. et al. [20] introduced the SPARC (Spectral Arc Length) metric, which calculates the Fourier transform of the arc length of a normalized motion velocity profile. This metric meets the validation criteria for calculating smoothness defined by [17,31], so it can be said that the SPARC metric allows for quantifying the intermittency of movement independently of its amplitude and duration [19]. In 2018, Becky Y. et al. [18] applied the SPARC smoothness metric to acceleration data from an IMU placed on the lower back of patients with Parkinson’s disease to determine whether SPARC provided helpful information allowing the researchers to characterize the gait objectively. They found that SPARC allows for the differentiation of gaits between medicated and unmedicated subjects. In 2021, García, F. V. [24] applied the SPARC metric to the angular velocities of IMUs placed on the torsos of patients with chronic stroke who presented mild to moderate or severe motor impairment. With SPARC, reduced smoothness in both the stance and sway was found in stroke subjects compared to the control group. Recently, in 2022, Montemurro R. [32] used the SPARC metric and the Log Dimensionless Jerk (LDLJ) when processing the linear and angular accelerations of three IMU sensors placed in the lumbar area and on the legs, respectively, in patients with multiple sclerosis, patients with craniocerebral trauma (Traumatic Brain Injury), and healthy adults. They determined that both metrics allow for differentiation between healthy adults and populations with neurological diseases. Likewise, they also differentiated between the two neurological populations [23,31]; however, in this study, the researcher only investigated the signals from an IMU placed on the torso, without segmenting the obtained signal based on the stride.

There is a lack of consensus on which signal to use, with some authors utilizing angular velocity while others utilize linear acceleration. Likewise, it has yet to be concluded which axis of acceleration or velocity the smoothness should be calculated against or if it is better to use the resultant. Moreover, most articles have only examined smoothness with IMUs placed on the torso, pelvis, or upper legs [33,34], along with other body regions such as the knee and foot. These authors have recommended using a single sensor at the level of the pelvis [35,36] to minimize intrusion and avoid altering the gait [37]. In contrast, algorithms developed to analyze the gait based on IMUs situated on the calf and foot are considered more accurate [38,39]. Besides, given that changes in acceleration are more significant in the distal parts, these areas requires further investigation.

Furthermore, some authors [32] have only calculated smoothness using the entire signal obtained from walking, without segmenting it into phases [36]. To overcome that limitation, the present work compares the calculation of smoothness of the complete gait signal against the signal segmented by gait phases using the LDLJ, SPARC, and Peaks Metric and identifies the best location for the IMU within a biomechanical model, which allows us to identify events and calculate the smoothness in phases. Also, the photogrammetry system is used as a reference to compare the acceleration and event detection with those obtained with IMU signals. The intention of using IMUs is to perform future measurements away from a specialized laboratory where the measurement conditions with the photogrammetry system must be very controlled. Implementing IMUs in each biomechanical segment responds to the need to know in which IMU location gait data can be obtained without affecting the gait that is determined, as they remain reliable and relevant. Applying different smoothness metrics to the acceleration signals allows us to determine which metric we can use to extract more exact information about what happens in the patient’s gait and thus reach a more precise diagnostic conclusion in the future. Thus, the main contributions are (1) defining a methodology for applying gait smoothness metrics; (2) determining the best location of the IMU using smoothness metric qualifications; (3) determining the differences between applying the smoothness metrics with the complete gait signal and with the signal segmented by phases; and (4) determining which of the metrics has the best variance based on the IMU position. We measure 20 healthy subjects to establish a methodology for and normal values of smoothness in gait using various types of measurement such as photogrammetry system, inertial measurement units (IMU) and an instrumented mat. The first method is a reference parameter for the measurement and correct identification of the primary events of the gait phases. The second involves two types of IMUs: the XSENS sensors allow us to obtain linear acceleration and angular velocity signals from each biomechanical segment; the VICON TRIDENT is a reference used to correlate the data obtained from the XSENS with that of the photogrammetry system. Finally, the instrumented mat is used to ensure that the gait measured in each patient is considered healthy or normal. The signals obtained with the IMUs placed in different lower limb parts were applied to the three smoothness metrics described later. Subsequently, statistical analyses of variance were conducted with the data obtained to determine which anatomical location provided the best information without physically interfering with the normal gait pattern of the subject based on the calculation of the smoothness metric that was most suitable for use, for more detailed information of the process see Appendix A. As a final result of this procedure, a comparison was made between executing the entire process using the continuous signal, that is, the complete signal of the gait from start to finish, and an alternative analysis using the segmented signal at each step or stride of the gait and during each phase of the gait (support/swing). The discussion includes points for improvement in this procedure and the developed analysis, issues that were analyzed and resolved during this research, and future work that is currently in progress and could be pursued.

## 2. Materials and Methods

Twenty healthy participants were recruited: twelve men and eight women, aged 24 ± 2.1 years; height 167 ± 7.68 cm; weight 73 ± 14 kg; right leg length 89.5 ± 4.56 cm; and left leg length 89.25 ± 4.44 cm, with no history of any gait-related pathology. That was based on the results of the functional ambulation profile (FAP) from the GAITRite, which averaged 98.39 points out of 100. A score above 95 indicated a normal gait. All test subjects provided written informed consent, which the institute’s ethics committee reviewed. The measurements were conducted in the Movement Analysis Laboratory of the Luis Guillermo Ibarra Ibarra National Institute of Rehabilitation in Mexico City.

The VICON photogrammetry system was used with 9 Vero cameras (v2.2 model, 1280 × 1024 resolution, max frame rate of 330 at 2.2 MP, 3.4 ms latency) and 6 Vantage cameras (v5 model, 5 MP resolution, max frame rate of 420 at 5 MP, latency of 4.7 ms) synchronized with VICON Nexus 2.12.1 software. Participants were fitted with 14 mm infrared light-reflective markers following the Plug-In Gait Lower Body AI model [9]. Likewise, four inertial sensors from the VICON Blue Trident [40] brand were placed, composed of an accelerometer for linear acceleration (low ±16 g and high ±200 g, sampling rate from 1125 Hz to 1600 Hz, sensitivity of 16 bits and 13 bits, triaxial both), a gyroscope for angular velocity (±2000°/s, sample rate of 1125 Hz, sensitivity of 16 bits, triaxial), and a magnetometer for direction/position (±5900 µT, sampling rate of 112 Hz, sensitivity of 16 bits, triaxial) [40] connected via Bluetooth to an iPad. At the same time, seven inertial sensors of model MTx by XSENS [41] were connected through a cable to the data-logger (XbusMaster [41]), which acquired data from the inertial sensors with a sample rate of up to 512 Hz, synchronized all seven IMUs, and sent the data via Bluetooth to a receiver located on a PC.

**Figure 1:**
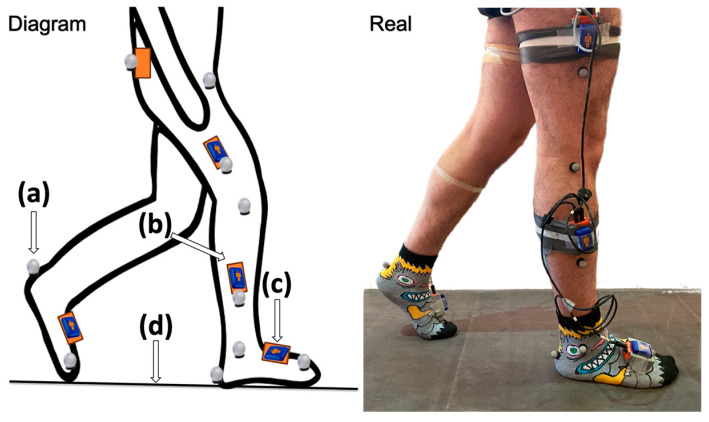
Placement of data collection methods. (**a**) In gray circles, placement of 14 mm VICON markers according to the Plug-In Gait Lower Body AI model. (**b**) In orange rectangles, the XSENS IMUs are placed symmetrically on the lower limbs: on the lateral part of the thigh and leg, the dorsum of the foot, and the posterior part of the hip. (**c**) In blue chips, TRIDENT VICON: IMUs are placed symmetrically on the side of the thigh, leg, and back of each foot. (**d**) GAITRite mat is 5.79 m long and 0.89 m wide.

The IMUs were placed following the biomechanical model proposed in [42]: one was positioned between the two posterior iliac, two were on the lateral parts of the thighs, two on the lateral parts of the legs, and finally, two on the dorsa of the feet, all with a pre-established orientation, as shown in Figure 1. Only four VICON Blue Trident sensors were placed: three on the right limb and just one on the left foot (see Figure 1), as the large number of sensors connected by Bluetooth meant there was a lag in the collection of data, so we decided to instrument only the right leg. For this, we decided to keep the VICON Blue Trident sensors, because the data are processed in the Nexus 2.12.1 software in such a way that the acceleration signals from the IMUs can later be synchronized with the axis-position signals from the cameras, allowing for the comparison of both signals and the design of a filter for the signal of the XSENS IMUs. This filter allowed the different acceleration signals to be compared and synchronized through the start-of-walk movement established as part of the measurement protocol described later in the article. According to the proposed model, the acceleration comparison points for the IMUs and the calculation by the VICON system are as shown in Table 1.

Once the markers and IMUs were placed on the participants, they were asked to walk in a straight line on the instrumented GAITRite walking mat [43], from which the spatiotemporal parameters and the functional ambulation profile (FAP) were obtained, to ensure that the gaits observed were normal. The participants walked seven times on the mat for a distance of 5.4 m, always starting with the right foot and moving in one direction, with a one-minute pause between each attempt. The data were stored in .xlsx files for later analysis. The XSENS was calibrated by placing the sensor on a measurement axis and performing a reset through the software.

The experimental data were processed, excluding each trial’s first and last step to omit the existing acceleration, deceleration, turning, and inactivity stages [37,44]. Attempts with a minimum of four strides were considered valid since that was the minimum necessary for calculating the spatiotemporal parameters [45]; the attempts that did not meet this condition were discarded, thus leaving a total of 51 trials per competitor. The rotation axes of the IMUs were considered as follows: *x*-axis = yaw, *y*-axis = pitch, and *z*-axis = roll [24,39]. The axis of the IMU that corresponds with the mediolateral axis of the leg producing the flexion–extension movement is ${yaw}_{foot}$; ${pitch}_{leg}$; ${pitch}_{thigh}$; ${roll}_{hip}$. The anteroposterior axis that produces the adduction–abduction movement remains ${pitch}_{foot}$; ${roll}_{leg}$; ${roll}_{thigh}$; ${pitch}_{hip}$. The longitudinal axis that makes the rotation movement remains ${roll}_{foot}$; ${yaw}_{leg}$; ${yaw}_{hip}$.

The data acquired from each system (VICON photogrammetry, VICON Trident, and XSENS), including linear acceleration and angular velocity on each axis, were taken and the resultants were calculated. They were derived twice: first, to obtain the Jerk, and second, to derive the double Jerk [29,46]. These data were re-sampled using the MATLAB function “Change Signal Sample Rate” [45] to homogenize the sample size of each trial at 100 Hz [47]. This was necessary because the VICON photogrammetry system returned a sampling frequency of 100 Hz, the VICON Trident IMUs operated at 250 Hz, and the XSENS IMUs at 59.6 Hz. Subsequently, a fourth-order Butterworth low-pass filter was applied to the IMU signals with a cut-off frequency of 5 Hz due to the walking frequency of 1 to 2 Hz [48]. This cut-off frequency does not hinder signal filtering.

Two events were identified in supporting us to recognize the phases of the gait within the strides: the initial contact or foot-strike and the take-off of the toes or foot-off. These were found in the processing of photogrammetry signals using the Nexus-VICON software 2.12. Once the trials were filtered, the events identified with photogrammetry were placed in all the IMU signals. With that information, an algorithm that automatically identifies gait events was developed in MATLAB App design (MathWorks, 2020^a^) so that photogrammetry could be dispensed with in future measurements; this algorithm also calculates the spatiotemporal parameters. Initially, the foot-off and foot-strike events were detected manually using the VICON system procedure; then, taking these temporal values as a reference, the signals obtained from each IMU were marked using this information, see Appendix A. With this, the acceleration axis component was identified, which allowed events to be recognized more efficiently. This resulted in the maximum angular velocity in the *y*-axis (pitch) of the IMU placed on the foot being used to detect the foot-off event, which indicates the beginning of a step and stride. The maxima of the linear acceleration in the *y*-axis (pitch) of the IMU placed on the foot was used to detect the foot-strike event, which signifies the end of a step and stride.

The smoothness metrics (SPARC, Peaks Metric, and LDLJ) described by Balasubramanian et al. (2015) [17] were applied to the signals automatically segmented by strides (5 ± 1 bilateral strides per gait). Although there is still no consensus on the appropriate metric to use in different tasks or with varying measurement technologies [49], we determined that it was appropriate to use these metrics. Normal healthy walking in adults is commonly two strides per second [48], so the cut-off frequency for the smoothness metrics was set at 10 Hz. It is important to emphasize that the spectral analysis of a signal follows the that less smooth movements are more complex in terms of their frequency composition. Therefore, an increase in negative values of the metrics indicates less smoothness of the movement. Likewise, adequate smoothness of movement has been associated with satisfactory balance scores (risk of falls) [50], motor control [51], and spatiotemporal gait parameters such as speed [12].

Equations (1)–(5) describe the different smoothness metrics used in this study. The Spectral Arc Length Metric (SPARC) is defined as the size of the Fourier spectrum curve of a movement’s velocity profile and is presented in Equations (1) and (2) [17].

(1) $$\lambda_{S}^{v}\left(V\right)≜-\int_{0}^{\omega_{c}}{\left[{\left(\frac{1}{\omega_{c}}\right)}^{2}+{\left(\frac{d\hat{V}\left(\omega \right)}{d\omega }\right)}^{2}\right]}^{\frac{1}{2}}d\omega$$

with

(2) $$\hat{V}\left(\omega \right)=\frac{V\left(\omega \right)}{V\left(0\right)};\;\;\;\;V\left(\omega \right)=\left|F\left({\left|\left|v\left(t\right)\right|\right|}_{2}\right)\right|$$

The authors of [17] suggest that SPARC, a semi-periodic signal of accelerations, should be used to measure gait signals. Alternatively, the Peaks Metric may be used, which is the number of local maxima in a movement’s velocity profile, presented in Equation (3) [17].

(3) $$NP≜-\left|\left\{v\left(t\right),\frac{dv\left(t\right)}{dt}=0\;and\;\frac{d^{2}v\left(t\right)}{dt^{2}}<0\right\}\right|$$

Finally, the Log Dimensionless Jerk Metric is the negative logarithm of the squared normalized total Jerk as presented in Equations (4) and (5) [18]. It is considered the most valid method since it adequately quantifies the deviations of smooth and coordinated movements [52].

(4) $$\lambda_{L}^{v}\left(v\right)≜-\ln\left(\frac{{\left(t_{2}-t_{1}\right)}^{3}}{v_{peak}^{2}}\int_{t_{1}}^{t_{2}}{\left\|\frac{d^{2}}{dt^{2}}v\left(t\right)\right\|}_{2}^{2}dt\right)$$

with

(5) $$v_{peak}≜\max{\left\|v\left(t\right)\right\|}_{2},t\epsilon \left[t_{1}{,}_{2}\right]$$

**Figure 2:**
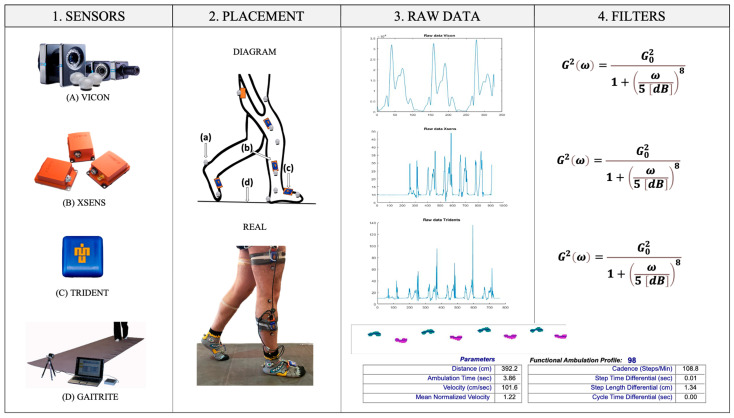
Diagram of the methodology (from left to right) implemented as part of the measurement protocol: data acquisition, data processing, and signal filtering. (1) Sensors indicate the four methods of obtaining data. (2) Placement, location of the sensors on the diagram, and the subject’s body for measurement. (**a**) In gray circles, placement of 14 mm VICON markers. (**b**) In orange rectangles, the XSENS IMUs. (**c**) In blue chips, TRIDENT VICON. (**d**) GAITRite mat. (3) Raw data and graphs of the unprocessed signals from each sensor detecting the right foot’s magnitude. (4) Filters describe the filter equation used for the raw data.

**Figure 3:**
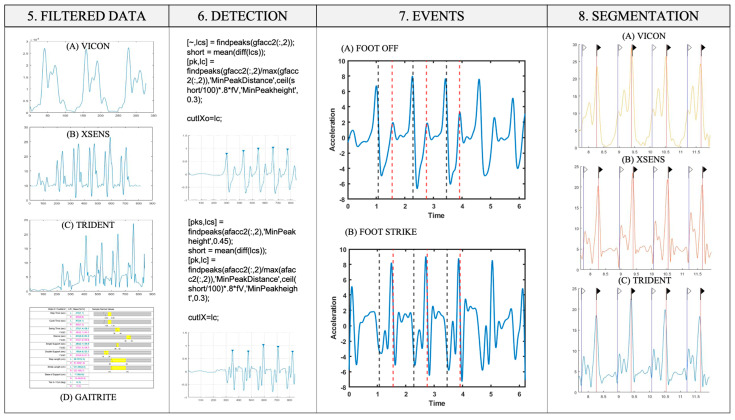
Diagram of the methodology (from left to right) implemented as part of the measurement protocol: the processed data from each sensor, the event detection algorithm, the events detected in the acceleration signals, and the signals segmented by the events marked in all the signals. (5) Filtered signals from all sensors consist of signals from the right foot’s magnitude. (6) The event detection algorithm is explained in MATLAB code. (7) The event detection algorithm is applied to each magnitude and component of the IMU signals, and each gear’s foot-off and foot-strike events are detected. (7A) The y component of the linear acceleration is shown. (7B) The y component of the angular acceleration is shown. (8) The events detected by the algorithm in each of the acceleration signals from each sensor are shown.

**Figure 4:**
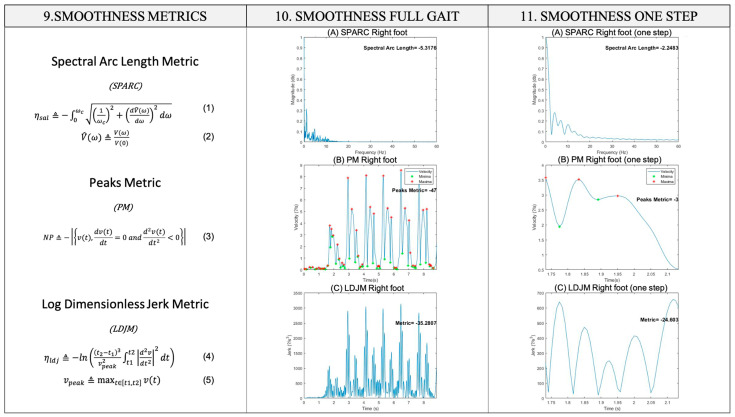
Diagram of the methodology (from left to right) implemented as part of the measurement protocol: equations of the smoothness metrics, application of the smoothness metrics to the entire gait signal, and application of the smoothness metrics to the segmented stride signals of each march. (9) Equations of the smoothness metrics (Equations (1)–(5)), are used to evaluate the motor gesture of walking. (10) Smoothness metrics are applied to the complete signal in its components and magnitude. (11) Smoothness metrics are applied to acceleration signals in their components, and magnitude is applied to the signal segmented by gait strides.

In all equations, v represents linear velocity, t denotes time, and w signifies angular velocity. For the analysis of the average results of the three smoothness metrics of each stride, the FAP [53] obtained from the GAITRite mat was considered based on the spatiotemporal parameters of the gait, where a value between 95 and 100 points denotes a normal gait [54]. These data were used to ensure that the measured gait was the most typically normal for healthy subjects. Figure 2, Figure 3 and Figure 4 present a complete outline of the methodology used in this article with each of its phases.

## 3. Results

### 3.1. Event Detection and Gait Segmentation

**Figure 5:**
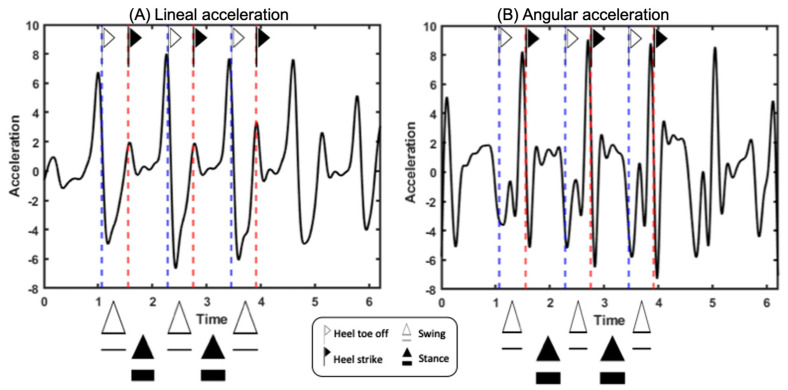
Both graphs show the events marked by the signal maxima to determine the foot-off and foot-strike events; the white flag indicates the detection of standing foot-offs, and the black flag indicates the detection of foot-strikes. White triangles indicate the signal zone between standing foot-off and foot-strike, indicating the swing phase. The black triangles indicate the signal zone between the foot-strike and the following standing foot-off, indicating the stance phase. (Left) graph (**A**) shows the linear acceleration signal on the *y*-axis (pitch), and (right) graph (**B**) the angular acceleration on the *y*-axis (pitch).

**Figure 6:**
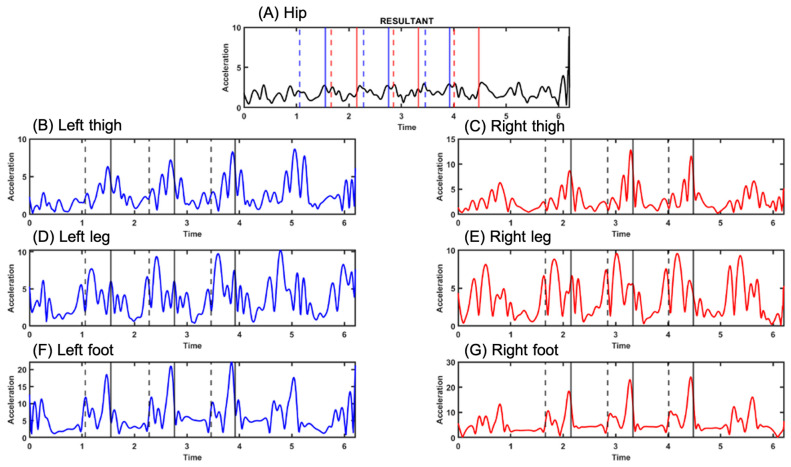
The XSENS IMUs obtained these seven signals. The solid line represents the foot-strike, and the dashed line represents the foot-off in each graphic.

Subjects walked an average of 3.88 m from initial contact to final contact, with an average duration of 3.43 s, cadence of 105.6 steps per minute, and velocity of 1.138 m/s. They took a total of six steps, with 38.4% of their time on the swing and 61.6% on the stance, and with 38.8% of their time in single support and 22.8% in double support. They had a step time of 0.56 s and a cycle time of 1.10 s, and they had a step length of 63 cm and a stride length of 127 cm. Once the gait events were identified, the signals from each of the IMUs were segmented into each segment of the biomechanical model, as can be seen in Figure 5. In Figure 6, The signals obtained from the XSENS located (A) in the back of the pelvis, and on the lateral parts of the (B) left thigh, (C) right thigh, (D) left leg, (E) right leg, (F) left foot, and (G) right foot. In Figure 6, signal (A), the solid blue line represents the left lateral foot-strike, and the dashed line represents the standing foot-off, and the red line indicates the same but for the right lateral foot-strike. The foot-strike and foot-off events are also marked on signals B, C, D, E, F, and G. The solid black line represents foot-strikes, and the dashed line represents standing take-offs. When the two methods for identifying events (VICON procedure vs. MATLAB algorithm) were compared, an error of less than 2% was obtained in each of the 20 subjects’ walking steps (≈666 measurements).

### 3.2. Assessment of the Smoothness of Gait

**Figure 7:**
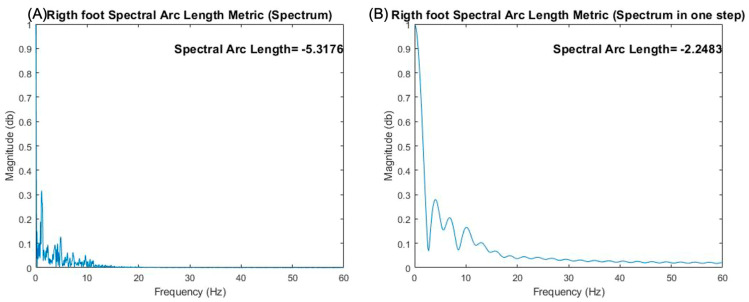
The graphs obtained from the SPARC metric in analyzing the entire gait (**A**) and the study of a stride with the SPARC metric in magnitude (**B**).

**Figure 8:**
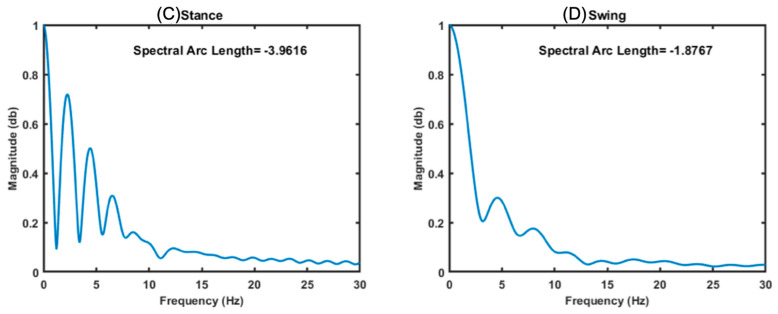
The smoothness in the SPARC metric in the stance phase on the left side (**C**) and in the swing phase on the right side (**D**) for a stride.

According to [32,36], the smoothness was calculated in all the signals obtained from the IMU, without segmenting, using the three equations described above, as can be seen in Figure 7A following the algorithm proposed by [32,36], where the analysis of several strides is shown, and Figure 7B using the same procedure, but applying a single stride. It can be observed that there is a difference between applying the smoothness algorithm to the entire gait signal and to a single stride. However, as our interest was in determining the smoothness behavior in each phase of the gait, once the signals were segmented, the same three algorithms were applied to them to obtain the smoothness of movement of each stride of both legs; see Figure 8, where Figure 8C shows the smoothness in the support phase, and Figure 8D shows the smoothness in the swing.

The metric’s result takes a negative value, with 0 being an ideal SPARC value where the movement is as smooth as possible. The lower the SPARC value, the greater the intermittency and the less smoothness calculated in the movement performed. Table 2 shows the average smoothness levels of the twenty subjects in the analysis of the yaw, pitch, and roll components and their magnitude in each of the segments in the full gait and according to the averages in the support and swing phases.

As in other studies [19], differences were sought between the variables based on the results of the three smoothness metrics obtained by testing the unsegmented signals against the analysis of a single stride. For this, we used Kruskal–Wallis analysis, because the data were non-parametric [24,53]. This one-factor analysis of variance showed a significant difference between the SPARC, PM, and LDLJ variables in the feet with $H_{\left({4.67e}^{-19}\right)}\;$ = 84.41, in the legs with $H_{\left({7.803e}^{-20}\right)}$ = 87.99, in the thighs with $H_{\left({6.905e}^{-21}\right)}\;$ = 92.84, and in the hips with $H_{\left({3.382e}^{-19}\right)}\;$ = 85.06; therefore, with the available data, the null hypothesis was rejected (that there would be no difference between the categories of the independent variable SPARC concerning the categories of the LDLJ and PM variables of stride and complete gait). The alternative hypothesis was accepted (that there would be a difference between the categories of the independent variable SPARC concerning the categories of the LDLJ and PM variables of stride and complete gait). The *p*-value was <0.00001, and the results were significant at *p* < 0.05, so the Kruskal–Wallis test showed a substantial difference between metrics.

Table 3 shows the results of the Kruskal–Wallis test comparing the smoothness metric in the gait without segmentation and segmented by stride. The green cells indicate a statistically significant difference between the two groups analyzed (*p* < 0.05). Otherwise, the cell is marked in red, for example, for LDLJ(C) vs. LDLJ(S).

Then, to the gait smoothness signals segmented with the foot-strike and foot-off events, to compare the groups in pairs and determine which were significantly different. The Bonferroni posthoc test revealed that the pairwise group comparisons of SPARC–PM and PM–LDLJ a *p*-value < 0.05. Therefore, it be assumed that these groups significantly different pairwise.

**Figure 9:**
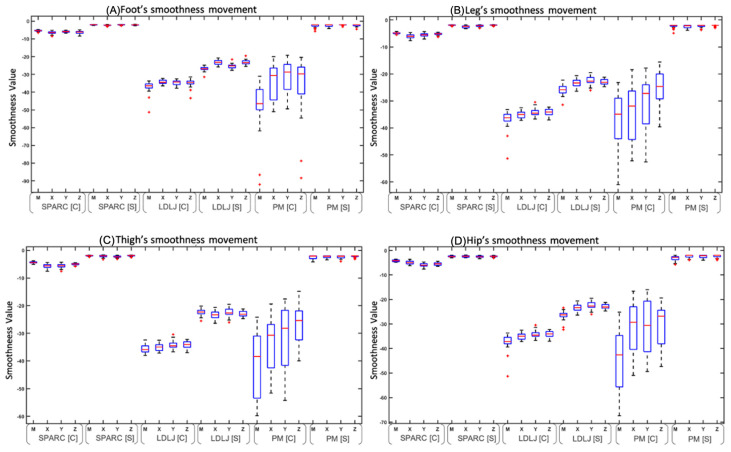
The graph shows the average (blue box) ±1 standard deviation (dashed line) of each smoothness metric (magnitude (M), yaw (X), pitch (Y), and roll (Z)) of the complete gait and the average per stride according to the IMUs placed on the feet, legs, thighs, and hips.

Subsequently, the variances obtained from the smoothness metrics were compared, as shown in Figure 9. This figure shows that the magnitude and components of the SPARC metric had the lowest standard deviation (SD) in the full gait and when segmented by stride compared to the other two metrics. On the contrary, in the same graphs, PM in the full gait has a very large SD. This analysis was repeated for the leg, thigh, and hip segments. As SPARC had the least variation [14], the rest of the analysis was only performed with the SPARC metric data.

Finally, a Kruskal–Wallis analysis was performed to find the optimal locations of the IMUs within the biomechanical model, only using the data obtained with the SPARC metric. The data from the IMUs located on the feet, legs, thighs, and hips were compared, taking into account the orientation of the IMUs. It was determined that on the *x*-axis (yaw), the IMUs located on the feet, legs, and hips were not significantly different. Still, they were different from the data of the IMUs situated on the thighs. On the *y*-axis (pitch), the IMUs located on the feet and legs did not have significant differences from one another, but they did compared to the IMUs on the thighs and hips. For the *z*-axis (roll), it was observed that the IMUs located on the feet significantly differed from all others. However, the IMUs on the legs and thighs did not differ significantly from one another. Finally, when analyzing the results, we found there were only significant differences in the IMUs on the hips. The final Kruskal–Wallis test results showed that the measurements performed on the feet with each smoothness metric differed. Concerning the objectives established in the introduction of this article, we achieved the following outcomes: (1) A methodology to follow for the correct measurement of the gait in healthy subjects was established, and we determined that acceleration signals can be measured using only IMUs without the need to have a photogrammetry system as a backup, which makes it possible to carry out future measurements outside this controlled environment. (2) The best locations of the IMUs were determined using this methodology, as the statistical analysis indicated differences between the measurements made by the three smoothness metrics in each of the anatomical locations, and that the location used does not affect or bother the subject being measured; in the same way, it allowed us to reach a conclusion about which metric best assessed the smoothness characteristic. (3) Segmenting the gait into its stance and swing phases and then applying the smoothness metrics to each of the biomechanical segments allowed us to perform a more in-depth analysis, which we recommend for future work on gait analysis since it showed that measuring the complete gait is not the same as segmenting it into its main phases, for a deeper investigation. (4) It was possible to determine which metric is the most recommendable based on the statistical analysis of variances between the locations of the IMUs.

## 4. Discussion

Previous research suggests that gait event detection is more accurate when using an IMU on a lower extremity (leg or foot) [55] compared to an IMU worn on the lower back or a thigh [7,39]. The results obtained in this work align with those findings; it was also observed that it is possible to calculate gait events in other anatomical positions. However, we found it simpler to do so with the signals from IMUs located on the legs or feet [34,38].

With the gait events identified as foot-strike and foot-off, the signals were segmented by strides, and the support and swing phases of the gait were identified, which made it possible to apply the smoothness metrics in these phases and determine their contributions. When using the smoothness metrics (SPARC, LDLJ, and PM), significant differences were found between the results with the complete signal (C) and the signal segmented by strides (S). Likewise, upon analysis, it was found that the SPARC metric applied to the complete signal and the segmented signal had the most significant sensitivity to the gait smoothness, unlike the findings of other studies [32,36]. Another insight we made was that implementing smoothness metrics less commonly used can lead to a new approach to gait analysis. The results also indicated that once the gait signal is segmented, it is essential to consider the anatomical locations of the IMUs, given that there were significant differences between the smoothness results in the different positions of the IMUs according to the biomechanical model proposed. The smoothness data of the IMUs located in the foot segment had the least variability recorded, so this was the segment in which the best results for smoothness were obtained; this is since the foot is the most distal segment, which experiences a more significant change in acceleration and deceleration compared to the other segments [56,57]. Some authors [34,39] suggest that placing the sensor on the waist is much less intrusive and propose that the minimal weight and volume of the IMU cannot alter the waist measurement. However, with the results from this work, we assert that placing two IMUs of the same size on each foot can also prevent interference and, therefore, modification of the subject’s gait.

It is essential to note that these results only represent the sample taken. Increasing the sample size would help generalize the findings produced in this study, and a more robust database would make the results more relevant.

A limitation of this study is that the gait data collection must be highly controlled to guarantee that the differences between the data are due to changes in gait and not any other factor, so if this methodology is transferred to a clinical environment, aspects such as the length of the walking corridor, the initial posture of the body, the foot with which one starts, and the support and placement of the IMUs must be taken into account. Nonetheless, evaluating the smoothness of a subject’s gait raises the possibility of thoroughly analyzing the subject’s control during the support and swing, in the context of their health condition, and relating it to mechanical situations, such as dynamic posture and the risk of falls [58,59], or to the pathology or physical condition of the patient. Future work will explore how the detection of gait smoothness changes during consecutive measurements can be related to pathologies, thus identifying changes in the gait due to disease progression, aging, or the treatment received.

## 5. Conclusions

A new model was adopted to analyze movement when walking, objectively quantifying the most important characteristics of the subjects’ gaits using inertial sensors and the latest motion capture technology, such as a photogrammetry system. The effectiveness of the proposed new-event detection system and the quantification of motion smoothness were evaluated, and both proved to be practical, precise, time-effective, and low in computational cost. These features underscore the system’s suitability for gait assessment or integration into rehabilitation/assist devices. Additionally, we determined that using the SPARC metric is the most appropriate, especially if one has the possibility of segmenting the gait into strides by any desired method [49].

Furthermore, in addition to these aspects, the system’s ability to estimate spatiotemporal gait parameters makes it ideal for use to quantitatively benchmark the human gait. The next steps to take are to (1) increase the sample size (including healthy patients), (2) generate a database, (3) compare the data with pathological gaits, and (4) establish correlations between the spatiotemporal parameters and smoothness of movement for healthy subjects and for those with pathological gaits, respectively.

## Figures and Tables

**Table 1:** Comparison points of the two systems’ acceleration calculations.

IMU	Photogrammetry System
Hip	hip segment (left anterior superior iliac spine LASI, right anterior superior iliac spine RASI, left posterior superior iliac spine LPSI, right posterior superior iliac spine RPSI)
Left and right thigh	markers on the left thigh (LTHI) and right thigh (RTHI), respectively
Left and right leg	markers on the left tibia (LTIB) and right tibia (RTIB), respectively
Left and right	foot segment (left ankle LANK, left heel LHEE, left toe LTOE, right ankle RANK, right heel RHEE, right toe RTOE), respectively

**Table 2:** Results of the magnitudes in SPARC, PM, and LDLJ.

SEGMENT *		RESULTANT																	
		SPARC						LDLJ						PM					
		STRIDE		SWING		STANCE		STRIDE		SWING		STANCE		STRIDE		SWING		STANCE	
		L	R	L	R	L	R	L	R	L	R	L	R	L	R	L	R	L	R
(1)	AVG	−5.401	−5.382	−2.071	−1.996	−3.592	−3.824	26.606	26.540	−26.743	−26.732	−28.109	−28.356	−5.624	−5.197	−2.419	−2.349	−3.921	−4.471
	±SD	0.638	0.720	0.176	0.127	0.872	0.691	1.989	2.837	0.862	0.717	1.411	0.632	13.234	12.706	0.659	0.423	0.842	1.714
(2)	AVG	−4.813	−4.746	−2.003	−1.898	−3.040	−3.147	24.380	26.540	−24.633	−25.442	−25.510	−26.589	−5.027	−4.797	−2.410	−2.306	−3.283	−3.530
	±SD	0.615	0.551	0.191	0.178	0.532	0.211	2.985	2.837	1.002	0.935	1.087	0.985	14.410	13.471	0.778	0.668	0.894	1.475
(3)	AVG	−4.492	−4.421	−2.020	−1.907	−2.339	−2.300	22.809	22.085	−22.561	−21.979	−23.639	−23.449	−4.474	−4.440	−2.428	−2.461	−3.513	−3.664
	±SD	0.718	0.602	0.245	0.131	0.362	0.248	2.606	2.930	1.186	0.935	1.087	0.985	12.514	15.847	0.514	0.556	1.151	1.348
(4)	AVG	−4.370		−2.432	−2.456	−2.653	−2.706	25.213		−21.623	−21.151	−22.290	−22.581	−4.428		−3.247	−3.027	−4.183	−4.466
	±SD	0.686		0.231	0.309	0.391	0.284	2.837		0.956	0.787	1.293	0.721	17.445		0.869	0.851	1.327	1.499

* (1) foot, (2) shank, (3) leg, (4) hip.

**Table 3:** Results of the Kruskal–Wallis analysis of the smoothness values of the metrics.

SMOOTHNESS METRIC		SEGMENT: FOOT		SEGMENT: LEG		SEGMENT: THIGH		SEGMENT: HIP	
Metric A	Metric B	A-B	*p*-Value	A-B	*p*-Value	A-B	*p*-Value	A-B	*p*-Value
SPARC(C)	SPARC(S)	−67.5	0.0	−70.475	0.0	−73.75	0.0	−53.65	0.002
LDLJ(C)	LDLJ(S)	−48.8	0.021	−58.65	0.002	−55.45	0.005	−35.1	0.136
PM(C)	PM(S)	−162.1	0.0	−143.375	0.0	−149.65	0.0	−112.625	0.0

The results were significant at *p* < 0.05.

## Data Availability

Data available upon request.

## Appendix A. Measurement Methods Purpose and Intended Use

Synchronization is not mentioned in the central part of this article since it was not required for the analysis. As explained in this paper, the methods of recording the acceleration signals are independent. Re-sampling techniques were performed as described in the researched literature. The three signals were re-sampled at the same sample rate as the photogrammetry system, which is the gold standard as a measurement reference; the VICON signal was manually marked with the foot-strike and foot-off events, following the basic procedure of the software, and the time stamps for each event were obtained. The IMU signals were filtered with an empirical filter that was found to make the signals identical to the signal obtained with the VICON cameras. Taking all this into account, as a rule, to start each measurement, the subject was told to start walking with their right leg, which gave us that marker as the start of the movement and the beginning of walking. With this and the three nearly identical signals, the signals could be synchronized.

1. VICON cameras: The gold standard gave us the first measurement reference. When we marked the gait events manually, it told us that there was no error. As this signal is from a more-than-proven system, we can use it again as the primary reference.
2. VICON Trident: This device obtained acceleration signals from the segments. From the beginning, it was determined that this was the easiest to synchronize with the cameras, as they are from the same brand.
3. XSENS: The IMU was adopted since it is low-cost and can be used in any other environment. It was proven that using seven sensors would not be a problem in synchronization since the system has been tested and used previously in different measurements.
4. GAITRite mat: When identifying the events and smoothness of the gait, it will always be easier to do this in a healthy gait that does not have other aspects that can hinder the measurement or alter the natural flow of the acceleration signal, and the use of this mat assured us with its measurement of the FAP (functional ambulation profile) that the gait was healthy by obtaining a score above 95 out of 100.
5. Three smoothness metrics: We recommend these three since there is no consensus on using a single smoothness metric. Although others are also used in the literature, we recommend these three.
